# Text Sentiment Analysis Based on Transformer and Augmentation

**DOI:** 10.3389/fpsyg.2022.906061

**Published:** 2022-05-13

**Authors:** Xiaokang Gong, Wenhao Ying, Shan Zhong, Shengrong Gong

**Affiliations:** ^1^School of Computer Science and Technology, Soochow University, Suzhou, China; ^2^School of Computer Science and Engineering, Changshu Institute of Technology, Suzhou, China

**Keywords:** sentiment analysis, social media, transformer, knowledge distillation, text augmentation

## Abstract

With the development of Internet technology, social media platforms have become an indispensable part of people’s lives, and social media have been integrated into people’s life, study, and work. On various forums, such as Taobao and Weibo, a large number of people’s footprints are left all the time. It is these chats, comments, and other remarks with people’s emotional evaluations that make up part of public opinion. Analysis of this network public opinion is conducive to maintaining the peaceful development of society. Therefore, sentiment analysis has become a hot research field and has made great strides as one of the hot topics in the field of natural language processing. Currently, the BERT model and its variants have achieved excellent results in the field of NLP. However, these models cannot be widely used due to huge demands on computing resources. Therefore, this paper proposes a model based on the transformer mechanism, which mainly includes two parts: knowledge distillation and text augmentation. The former is mainly used to reduce the number of parameters of the model, reducing the computational cost and training time of the model, and the latter is mainly used to expand the task text so that the model can achieve excellent results in the few-sample sentiment analysis task. Experiments show that our model achieves competitive results.

## Introduction

In today’s era of extremely complex social networking, the number of Internet users in China has increased dramatically. As of 2021, the number of domestic Internet users will reach 989 million (Cnnic, 2021). Such a huge scale that it involves a wide range of fields, such as shopping, games, communications, and video. In all these areas, there is a huge amount of data and information left behind, and these data and information have formed a certain tendency of online opinion on the Internet, which is both positive, negative, and neutral. Many companies can recommend products based on users’ comments and preferences, thereby increasing product sales; while some undesirable emotional messages can cause social unrest, such as donation scams, which can lead to heated debates among internet users. For the textual information formed by some remarks on the Internet, if sentiment analysis can be carried out in a certain way, the chaotic information can be extracted by certain means, and then differentiated according to different categories to form useful information for the society, which can ultimately guide the normal development of society is a direction worthy of study (Zhang et al., 2020).

To solve the above problems, deep learning plays an important role. Deep learning (LeCun et al., 2015) is to learn the inherent laws and representation levels of sample data, and the information obtained during these learning processes is of great help to the interpretation of data, such as text, images, and sounds. Its ultimate goal is to enable machines to have the ability to analyze and learn like humans, and to recognize data, such as words, images, and sounds. Deep learning is a complex machine learning algorithm that far outperforms previous related technologies in speech and image recognition.

Deep learning requires a large amount of labeled data to get excellent results, and the need for large amounts of labeled data is one of its most fundamental shortcomings. When the labeled data are limited, supervised deep learning models are tended to suffer from overfitting (Hawkins, 2004). The strong reliance on labeled data limits the practical use of deep learning models, due to the need for a large amount of time and money to obtain enough labeled data. As a result, semi-supervised learning (SSL; Van Engelen and Hoos, 2020) has received much attention, because it is one of the most promising paradigms of leveraging unlabeled data to address this weakness (Chawla and Karakoulas, 2005).

The current NLP technology is witnessing a revolution in pre-trained self-supervised models. These models often have hundreds of millions of parameters. Among these models, BERT (Devlin et al., 2018) is one of the most representatives. These models show substantial improvement in results on previous tasks. However, as one of the largest models in the history of natural language processing, the BERT model has a large number of parameters, large size, and high latency. These drawbacks prevent the model from running on devices with limited computing power or taking too long to run. In some real-time scenarios, the long response times would outweigh the benefits in comparison to the degree of improvement in accuracy. Therefore, we investigate reducing the number of parameters of the model while retaining its accuracy, thereby reducing the computational cost and response time required to run the model. This paper investigates how to reduce the parameters of the model. It is found that good results can be achieved using knowledge distillation and text augmentation for text tasks. Experimental results show that the accuracy of text sentiment classification can be improved with a small number of samples.

The contributions of this paper are summarized as follows:

1. We propose a text sentiment classification model based on the Transformer mechanism, which combines knowledge distillation and text augmentation methods to improve the accuracy of sentiment classification in the few-shot labeling task.
2. The model reduces the number of parameters based on the Transformer mechanism by means of knowledge distillation and uses the method of text augmentation to solve the problem of low accuracy for the few-sample task.
3. We conduct experiments on two public corpora and compare with different state-of-the-art methods to verify the performance of the model.

## Related Work

### Neural Language Model for NLP

Sentiment analysis is the classification of emotions and attitudes in subjective texts, and the main methods are machine learning and deep learning. From the perspective of machine learning, the text sentiment analysis method based on machine learning (Lin and Luo, 2020) needs to use a corpus to train a classification model. For example, Yan et al. (2020) used the bag-of-words model to classify text data and considered Word2Vec to establish a new word vector; in the ensemble algorithm Naive Bayes and SVM (Tiwari and Mallick, 2016), both the precision and recall rate were improved. From the perspective of deep learning, in recent years, deep learning technology has made great progress in processing text information-related tasks. The neural network structure has achieved remarkable results in text classification. The neural network structure is used to construct different neural network algorithms, such as CNN, bidirectional LSTM, text CNN, and so on. Chen (2015) first proposed to apply CNN to text orientation analysis, and obtained good results; on the basis of Chen’s analysis, Conneau et al. (2016) proposed a VDCNN model by using a deep convolutional network method. The subsequent rise of pre-trained models, which have led to major breakthroughs in the field of NLP, The BERT model proposed by (Vaswani et al., 2017) is a better improvement in terms of evaluation indicators. Devlin et al. (2018) mainly introduced the practical value of the BERT model, and obtained good research results on 11 natural language processing tasks.

One of the major breakthroughs in the history of natural language processing is the attention mechanism. The attention mechanism is proposed to solve long-term dependency problems of models that use a single context vector compressing every input from previous time steps. The attention mechanism makes it possible to capture the global semantics in the texts. Because the attention mechanism allows the model to take hidden states from multiple time steps as input and calculate the importance of the input regarding the current time step. Transformer serving as the core architecture of the attention mechanism has been widely used for language models (Han et al., 2021) to capture complex linguistic patterns. These pre-trained language models work well on various NLP tasks; however, it is accepted that these pre-trained models rely on large-scale data training and have high requirements for the quality of the data set.

### Pre-training and Fine-Tuning Framework

Neural network methods generally start with random initialization of model parameters and then train the model parameters using optimization algorithms, such as back propagation and gradient descent. Before the advent of pre-training techniques, the application of neural network-based deep learning in NLP faced the following problems: firstly, deep learning models at this time were not complex enough, and simply stacking neural network layers did not bring more performance gains; secondly, data-driven deep learning models lacked large-scale annotated data, and manual annotation was too expensive to drive complex models. Therefore, pre-training techniques based on knowledge augmentation, migration learning, and multi-task learning are gradually being given more attention by scholars.

The pre-training and fine-tuning framework have achieved great success in recent years, especially in the NLP field, and has been applied to a series of NLP tasks. Howard and Ruder (2018) proposed to pre-train a language model on a large corpus and fine-tune it on the target task. Such a model uses some novel techniques like gradual unfreezing and slanted triangular learning rates. Encouraged by the good performance of the pre-trained models, researchers get excellent performance even with small amounts of labeled data. Pre-trained models are often applied to different objectives, such as language modeling and masked language modeling. With the increase in the training data size, the performance of pre-trained models is also improved (Araci, 2019; Rezaeinia et al., 2019).

### Data Augmentation for Language Data

Data augmentation is a technique that can increase the size of a data set. Many researchers work on data augmentation, mainly in the field of computer vision, speech, etc. Compared to these, there is less research on data augmentation in the field of text and there is no standard method yet. The commonly used method is to use a dictionary or thesaurus or database of synonyms to make word replacements. In a situation where there is no dictionary, the other way is to use distributed word representation for finding similar words. A method belonging to this is called synonym augmentation. In fact, the best way to augment is to artificially change the wording of the language, but the cost of this method is too expensive. Therefore, the most option in data augmentation for most research is to replace words or phrases with their synonyms. For example, the most popular open-source lexical database for the English language is WordNet (Fellbaum, 2010). Another method is called semantic similarity augmentation, it uses distributed word representation, namely, word embedding, one can identify semantically similar words. This approach requires either pre-trained word embedding models for the language at hand or enough data from the target application to be able to build the embedding model. Its advantage is that no additional dictionaries are required to find synonyms. And the last method is back translation, its process is translating words, phrases, or texts into another language, namely, forward translation, then translating the results back into the original language, this is called back translation (Edunov et al., 2018).

Mixup augmentation (Guo, 2020) creates new training examples by drawing samples from the original data and combining them convexly, usually, the samples are two or even more, it combines the data both in terms of the input and output. It takes pair of samples from the initial data set and sums both the input and output. The main idea of the mixup is a sample. Given two labeled data ($x_{i},y_{i}$) and ($x_{j},y_{j})$, where *x* is the input vector and *y* is the one-hot encoded labels, the algorithm creates training samples by linear interpolations:


(1) $$\hat{x}=\lambda x_{i}+\left(1-\lambda \right)x_{j}$$

(2) $$\hat{y}=\lambda y_{i}+\left(1-\lambda \right)y_{j}\;$$

Where λ∈[0,1], and λ∼Beta(α,α)λ=max(λ,1−λ) in which *α* is the hyper-parameter to control the distribution of λ. It is best suited for learning models that use the cross-entropy loss and change the input. Augmentation by mixup can be done on text representation for text problems. as such we can use mixup with bag-of-words models, word embeddings, and language models.

### Knowledge Distillation

Deep learning has achieved incredible performance in numerous fields, including computer vision, speech recognition, natural language processing, and more. However, most models are too computationally expensive to run on mobile or embedded devices. Therefore, the model needs to be compressed, and knowledge distillation (Gou et al., 2021) is one of the important techniques in model compression. Knowledge distillation adopts the Teacher–Student mode: the complex and large model is used as the teacher, the student model structure is relatively simple, and the teacher is used to assist the training of the student model. The teacher has strong learning ability and can transfer the knowledge it has learned to relatively weak learning ability. The student model, in order to enhance the generalization ability of the student model. The complex and cumbersome but effective Teacher model is not online, it is simply a mentor role, and the flexible and lightweight Student model is really deployed online for prediction tasks.

Vanilla Knowledge Distillation is simply the learning of lightweight student models from the soft targets output by the teacher model. However, when the teacher model becomes deeper, learning the soft targets alone is not enough. Therefore, we need to acquire not only the knowledge output from the teacher model, but also other knowledge that is implicit in the teacher model, such as output feature knowledge, intermediate feature knowledge, relational feature knowledge, and structural feature knowledge. The four forms of knowledge distilled from the student’s problem-solving perspective can be compared to the following: output knowledge provides the answer to a problem, intermediate knowledge provides the process of solving a problem, relational knowledge provides the method of solving a problem, and structural knowledge provides the complete body of knowledge.

## Materials and Methods

In this section, we have made improvements to the Mixup method. Mixup can be interpreted in different ways. On the one hand, the Mixup method can be viewed as a data augmentation approach that creates new data based on the original training set. On the other hand, it enhances the regularization of the model. Mixup was first designed for images tasks; thus, mixup was demonstrated to work well on continuous image data; however, it is challenging to extend mixup from image to text, since it is infeasible to compute the interpolation of discrete tokens. To overcome this challenge, we propose a novel method that expands on the original text and interpolates in the hidden space. Inspired by the excellent performance of the pre-trained models like Bidirectional Encoder Representations from Transformers (BERT), we use a multi-layer model to encode the given text to get the semantic representations, in this process; we expand the original text and apply interpolations within hidden space as a data augment method for text. For an encoder with L layers, we choose to mix up the hidden representation at layer K.

**Figure 1:**
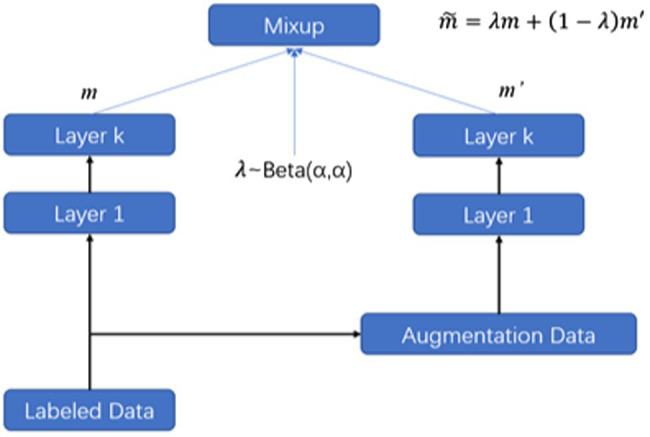
Mixup augmentation text.

As shown in Figure 1, we first use text augmentation techniques to expand the labeled data size, such as EDA, back translation, and Word2vec-based (learned semantic similarity) augmentation, and then, we compute the hidden representation in the bottom layers and mixup the hidden representation at layer m, and feed the interpolated hidden representations to the upper layers. In mathematical expressions, we denote the layer m in the encoder network as $mixup_{m}\left(.;\theta \right)$, thus the hidden representation of the layer m can be computed as $hidden_{m}=mixup_{m}\left(hidden_{m-1};\theta \right)$. For the text samples$\;X_{i}$ and its augmentation $X_{j}$ define the layer 0 as the embedding layer, for example, $h_{0}^{i}=W_{E}X_{i}$ and the hidden representation of the two samples from the lower layers and the mixup at layer k and forward passing to upper layers are defined as follows:


(3) $$h_{l}^{i}=mixup_{l}\left(h_{l-1}^{i};\theta \right),l\in \left[1,m\right]$$

(4) $${\hat{h}}_{m}=\lambda h_{m}^{i}+\left(1-\lambda \right)h_{m}^{j}$$

(5) $${\hat{h}}_{l}=mixup_{l}\left({\hat{h}}_{l-1};\theta \right),l\in \left[m+1,L\right]$$

(6) $$MATEXT\left(X_{i},X_{j},mixup\left(.;\theta \right),\lambda ,m\right)={\hat{h}}_{L}$$

We use an encoder model mixup(.;θ), Equation (3) represents the mixup method before the fusion layer *m*, where θ represents the remaining parameters in the model. Equation (4) expresses the calculation method in the mixed layer. Equations (5, 6), respectively, express the operation above the mixed layer and the whole mixing method. MATEXT combines the original text and its simple augmentation text as input and interpolates textual semantic hidden representations as a type of data augmentation. Compared to the mixup (Zhang et al., 2017) defined in the augmentation on text representation. In the experiment, we sample the mix parameter λ from a Beta distribution for every batch to perform the interpolation:


(7) λ~Beta(α,α)

(8) λ=max(λ,1−λ)

In the equation, α is the hyper-parameter to control the distribution of λ. In MATEXT, different from the original mixup, we share the label of the original text and its augmentation text.

### Data Augmentation

We use Easy Data Augmentation (EDA) technique (Wei and Zou, 2019) which consists of four different text editing operations: (1) Synonym replacement: N-words are randomly selected from the sentence and replaced with one of its synonyms chosen at random. (2) Random noise injection: N-words are randomly selected from the sentence and a single character of each word is replaced with a random alphabetical character. (3) Random swap: we randomly choose two words in the sentence and swap their positions and repeat N times. (4) Random deletion: N-words from the sentence are randomly chosen and removed from the sentence.

The value *N* is depended on the length of each sentence. In our experiment, the *p* is set 0.1 and calculated p×len(sentence), where the number words in the sentence is used as a length of the sentence. Rounded up value of p×len(sentence) is used as the value of N.

In addition, Back translations are also a common data augmentation technique and can generate diverse paraphrases while preserving the semantics of the original sentences. And Word2vec is another robust augmentation method that uses a word embedding model trained on the public data set to find the most similar words for a given input word, which is called Word2vec-based (learned semantic similarity) augmentation (Budhkar and Rudzicz, 2018). Table 1 shows some examples of text augmentation.

**Table 1:** Example of text augmentation.

Operation	Text
Origin	The film has several strong performances.
Synonym replacement	The film has several solid performances.
Random noise injection	The film has several strong ha performances.
Random swap	Several film has the strong performances.
Random deletion	Film has the strong performances.
Back translation	The film has several strong performances.
Word2vec-based	Another movies have not two strength showings.
Contextual-wordaug	However, the animated film usually has several strong performances.

### Knowledge Distillation

**Figure 2:**
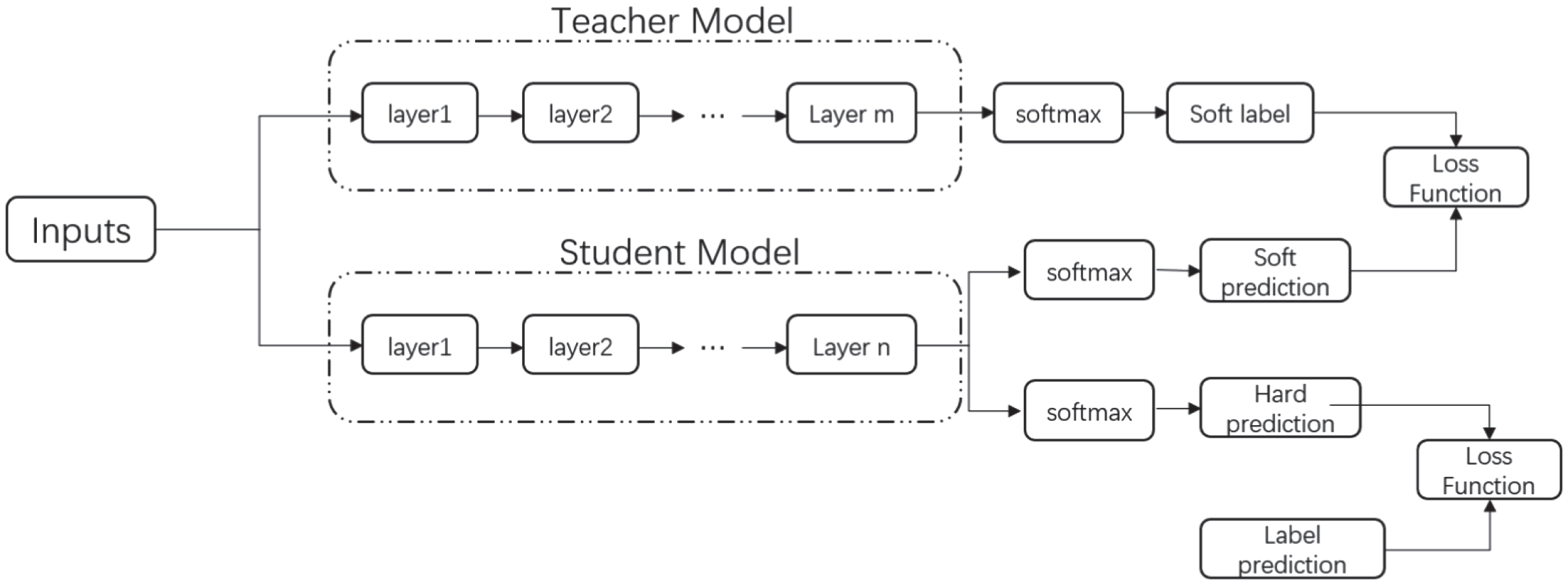
Knowledge distillation steps diagram.

The main purpose of this chapter is to reduce the parameters of the current mainstream BERT model through the method of knowledge distillation. We study the knowledge distillation under a limited amount of labeled data and a large amount of unlabeled data. With sufficiently accurate teacher models and large amounts of distilled unlabeled data, using Bi-LSTM and RNN as encoders as student models can greatly reduce the parameter space and perform on par with large pre-trained models. The distillation technique is mainly divided into two parts, first using a fine-tuned teacher model trained with labeled text data to automatically label a large amount of unlabeled data and then using the augmented data to train a student model with a supervised cross-entropy loss. In the second part, we used the logarithmic and internal representations from the transformer mechanism to generate training student models on unlabeled data, using different training schedules to optimize different loss functions, and experiments proved that this partial distillation can further regularize these models to improve performance. The specific steps are shown in Figure 2.

The definition of the loss function is shown in following formula:


(9) $$L=\alpha L_{soft}+\beta L_{hard}$$

The soft loss function is for the teacher model to label the unlabeled data and then train the student model, while the hard loss function is for the student model to predict the label and take the cross-entropy of the labeled data, because there may be different probability distributions of different categories in the training data. Balanced, a reasonable distribution of soft labels and hard labels, that is, the *T* value in the formula can improve the generalization ability of the model.


(10) $$L_{soft}=-\overset{N}{\underset{j}{\sum }}p_{j}^{T}\log\left(q_{j}^{T}\right)$$

(11) $$p_{i}^{T}=\frac{\exp\left(v_{i}/T\right)}{\sum_{k}^{N}\exp\left(v_{k}/T\right)}$$

(12) $$q_{i}^{T}=\frac{\exp\left(z_{i}/T\right)}{\sum_{k}^{N}\exp\left(z_{k}/T\right)}$$

(13) $$L_{hard}=-\overset{N}{\underset{j}{\sum }}c_{j}\log\left(q_{j}^{1}\right)$$

(14) $$q_{i}^{1}=\frac{\exp\left(z_{i}\right)}{\sum_{j}^{N}\exp\left(z_{j}\right)}$$

Among them, *T* is the temperature coefficient, which is used to control the softening degree of the output probability. It is easy to see that Equation (11) represents the class probability of the network output Softmax when *T* = 1. When *T* is positive infinity, Hinton et al. (2015) show in their paper that Equation (11) represents the logical unit of the network output at this point. v*_i_* and z*_i_* are both generated by the neural network using the softmax layer to generate class probabilities. The output layer converts the logarithm of z*_i_* calculated by each class into probabilities q*_i_* and pi by comparing z*_i_* and other logical probabilities. From Jiao et al. (2019), it is known from experiments that knowledge distillation from the transformer to the non-transformer framework will limit the knowledge learnable from the teacher model due to the incomparability of the parameters of the intermediate layers, due to the transfer of knowledge to smaller models, the effect is not good, and the knowledge distillation transferred to the same transformer framework, due to the interoperability of the middle layer, the parameters can be used, and the effect is better than the former because the reduction of the parameter amount will also reduce the running time in the actual processing process and computing power costs.

**Figure 3:**
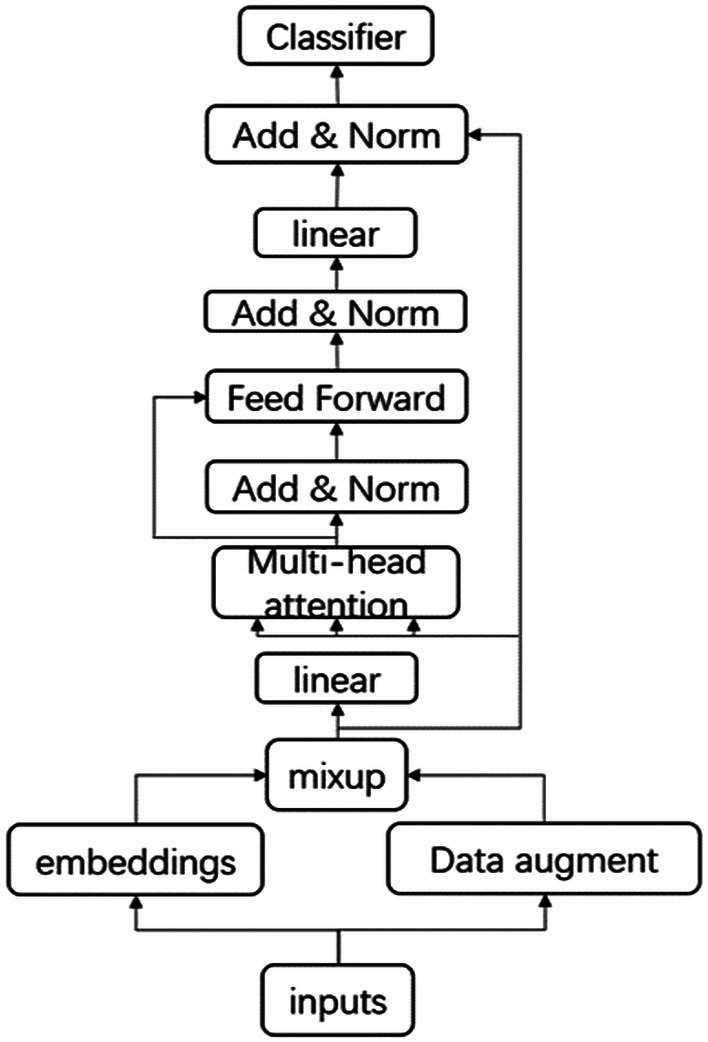
Model structure diagram.

The model structure we designed is shown in Figure 3. The input text data are expanded and then mixed in the transformer layer before training and classification. In addition to the traditional modules, the model is mainly divided into four parts, namely, the data augmentation part, the mixed layer, the linear layer, and the stacked module.

The mixup and text augmentation modules have been shown in the two vignettes above, and the linear layer in Figure 3 can also be referred to as the bottleneck layer. We have introduced this linear layer into the model for two reasons: on the one hand, this bottleneck layer allows for the scaling of the input and output sizes to 512, facilitating subsequent knowledge transfer and model training; on the other hand, the choice of a linear layer over an activation function prevents the non-linearity from corrupting too much information, while the activation function may filter out much useful information.

The addition of the bottleneck structure to the transformer layer causes the balance between the multi-head attention mechanism and the feedforward neural network to be broken, that is, the feature mapping of the input and output through the latter two will be different through the bottleneck layer, which can cause many problems. The overall structure of the multi-head attention mechanism allows the model to jointly process information in different subspaces, while the feedforward neural network increases the non-linearity of the model. In the original BERT model, the ratio of the multi-head attention mechanism to the feedforward neural network is 1:2. However, with the addition of the bottleneck structure, the input to the multi-head attention mechanism will come from a wider feature map, that is, the size of the modules between each other, while the input to the feedforward neural network will come from a narrower bottleneck, that is, the size of the modules. These two changes lead to the inclusion of more parameters in the multi-headed attention mechanism. To address this issue, stacked layers, that is, stacked feedforward neural networks, were introduced to rebalance the relative relationship between the multi-headed attention mechanism and the feedforward neural network. Size, and in this chapter, we use four stacked feedforward neural networks for balancing.

## Experiment

### Data Set Introduction

This chapter uses two data sets to analyze the model from multiple perspectives. These data sets are AG News Corpus, The Stanford Sentiment Treebank (SST) data set, and AG News Corpus with 496,835 items in four categories and more than 2,000 News articles from a news source, the data set has title and description fields, and each category has 30,000 training samples and 1,900 test samples, of which we randomly select 10,000 samples for training. The SST data set is a sentiment analysis data set released by Stanford University. Its essence is a sentiment classification task, mainly for the sentiment classification of movie reviews. Therefore, SST belongs to single-sentence text classification. We selected SST-2 for the two classification task.

### Model Settings

In order to compare the prediction effect of the model, we compared the results of multiple models on multiple tasks and compared the accuracy of multiple models on the classification task, in which a BERT-based-uncased tagger was used to tag the text, and use this model for text encoding. In order to reduce the parameters of the original BERT model, we designed the above model to change the effect of the student model by adjusting the ratio of the multi-head attention mechanism and the feedforward neural network, as shown by the experiments in Mobilebert. The model performance will peak when the ratio between the two is 0.4 ~ 0.6, so we choose a ratio of 0.5. In addition, the maximum sentence length is 256. For sentences that exceed the length, we keep the first 256 characters, the learning rate is set to 5e-5, and the batch size is 32. Due to the difference in the number of training samples, we set the saving step in the different numbers of samples. When the number of trains is the maximum value, please refer to Table 2 for detailed parameters.

**Table 2:** Experimental parameter table.

Hyperparameters	Value
The maximum length	256
batch size	32
Learning rate	5e-5
epoch	5
Save step	2,105

### Baselines

We tried to compare a number of different classification models. The following are the model names and their brief introductions.

#### BERT

This model is one of the current mainstream models; we use the already trained BERT-based-uncased model and fine-tune it for classification tasks.

#### ALBERT

This model is a simplified version of the BERT model. It is transformed on the basis of the BERT model and reduces a lot of parameters, which greatly improves the speed of model training and model prediction, and the effect of the model will only be a slight decrease (Lan et al., 2019).

#### MobileBert

This model is a compressed version of the BERT model (Sun et al., 2020). It uses knowledge distillation to improve based on BERT. Its scale is reduced by 3–4 times and the speed is increased by 4–5 times. Compared with the original knowledge distillation model, the model prunes parameters while retaining most of the accuracy.

### Result

This chapter conducts comparative experiments on three data sets, namely, the SST data set and the AG news corpus. Based on these three data sets, experiments are conducted with different data scales to test the effectiveness of the model; the results are shown in Table 3.

**Table 3:** Performance [test accuracy(%)] comparison with baselines.

Data sets	Model	100	500	1,000	5,000	All
AG NEWS	BERT	64.99	67.42	84.93	**89.93**	**93.38**
	ALBERT	60.14	76.45	84.06	89.64	92.06
	MobileBERT	65.05	75.88	84.48	89.47	92.74
	Our Method	**80.18**	**84.01**	**85.14**	89.68	93.28
	Model	20	100	500	1,000	All
SST	BERT	50.92	69.72	73.27	83.03	**92.94**
	ALBERT	48.81	63.87	81.19	83.14	92.42
	MobileBERT	50.91	55.28	81.65	84.17	91.28
	Our Method	**78.21**	**82.35**	**84.63**	**85.21**	90.03

Bold values indicate the best performance of different models on the same dataset.

As can be seen from the results in the table, we have cut the data sets and cut different scales on the different corpus. It can be seen that when the amount of data is sufficient, BERT The results obtained by the model are the best, but since they are all transformer-based models, the difference between the final results is not large. It is easy to see that the method given in this paper can achieve better results in the case of a small number of samples. For example, in the case where the number of labeled labels in the AG News data set is less than 1,000, the method given in this paper has achieved relatively good results. The excellent results, especially when there are only 100 labeled data are greatly improved compared to the other methods. When MobileBERT does not introduce the mixing layer and the data enhancement layer, it can be seen from the above table that the results achieved by the same type of data set and the same data scale are lower than those achieved by other models.

**Figure 4:**
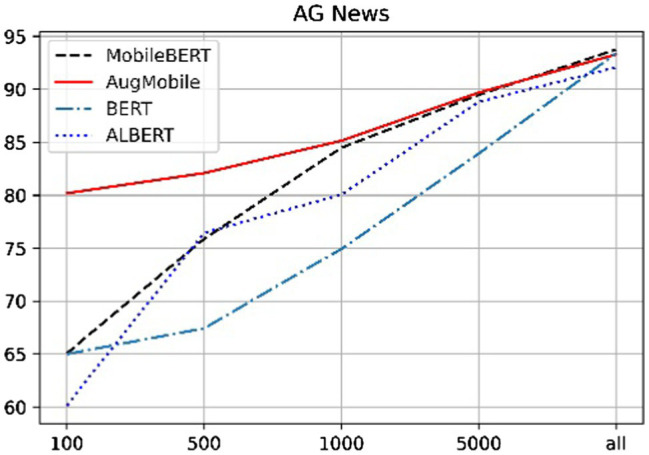
Performance [test accuracy (%)] on AG News.

**Figure 5:**
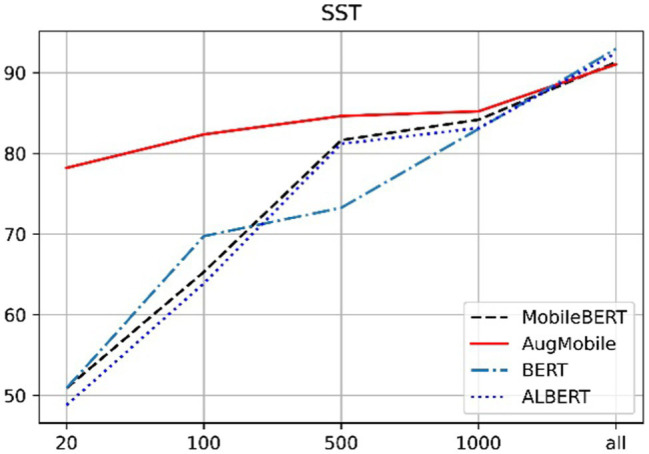
Performance [test accuracy (%)] on Stanford Sentiment Treebank (SST).

Figures 4, 5 can clearly show that when the sentiment analysis data set and the news data set are classified, especially when the number of labeled data is less than 100, our method has an improvement of about 20% compared with the other methods. This is due to the small amount of training data and the extreme demands of each transformer-based model for training data, both of which lead to problems, such as overfitting of the trained model, resulting in poor results. The method proposed in this paper is when the data scale is small, the method of text expansion is provided, which increases the scale of training data to a certain extent and thus has better results than other models. With the increase of data scale, the results of each model have a tendency to approach each other. When the data size is greater than 1,000, the difference between the results of each model is small. Similar to the principle of a small amount of training data, when the training data reach a certain size, the difference between the training results of each model is not large. The advantage of the method proposed in this paper is that our results are excellent when used in a small amount of labeled data, and also have good results in large-scale data.

## Conclusion

The purpose of text sentiment analysis is to analyze and study the large amount of comment information on the web and provide a relevant basis for the authorities to rectify the online environment. To alleviate the reliance on annotated data, a text sentiment classification method that combines data augmentation techniques and Transformer is proposed. Through experiments on two benchmark text classification data sets, the reliability and efficiency of text sentiment classification are fully weighed, and the reliability and efficiency of text sentiment classification are greatly improved, which also has great advantages compared with other state-of-the-art research. We plan to explore the effect on tasks outside the experimental data set, such as other real-world scenarios with limited labeled data.

## Data Availability Statement

Publicly available data sets were analyzed in this study. These data can be found at: https://gluebenchmark.com/tasks.

## Ethics Statement

The individual(s) provided their written informed consent for the publication of any identifiable images or data presented in this article.

## Author Contributions

XG was responsible for designing the framework of the entire manuscript, from topic selection to solution to experimental verification. All authors contributed to the article and approved the submitted version.

## Funding

This work was supported by the Humanities and Social Sciences Foundation of the Ministry of Education under Grant 18YJCZH229, and in part by the 13th Five-Year Plan Project of Educational Science in Jiangsu Province under Grant X-a/2018/10, and the National Natural Science Foundation of China (61972059).

## Conflict of Interest

The authors declare that the research was conducted in the absence of any commercial or financial relationships that could be construed as a potential conflict of interest.

## Publisher’s Note

All claims expressed in this article are solely those of the authors and do not necessarily represent those of their affiliated organizations, or those of the publisher, the editors and the reviewers. Any product that may be evaluated in this article, or claim that may be made by its manufacturer, is not guaranteed or endorsed by the publisher.
